# Characterization and Comparison of Polymer Melt Fluidity Across Three Ultrasonic Plasticization Molding Technologies

**DOI:** 10.3390/polym17192576

**Published:** 2025-09-24

**Authors:** Shiyun Wu, Jianjun Du, Junfeng Liang, Likuan Zhu, Jianguo Lei

**Affiliations:** 1 School of Mechanical Engineering and Automation, Harbin Institute of Technology (Shenzhen), Shenzhen 518055, China; wusy626@163.com (S.W.);; 2 College of Mechatronics and Control Engineering, Shenzhen University, Shenzhen 518061, China

**Keywords:** ultrasonic plasticization, ultrasonic pressing, microstructure formation, polymer melt fluidity, template microstructure damage

## Abstract

The influence of axial ultrasonic vibration (the dominant vibration mode) on the filling behavior of polymer melt in microcavities and its effect on microstructure formation remains inadequately understood. Based on the plasticization location and the extent to which the microcavity is covered by the ultrasonic sonotrode action surface, existing ultrasonic plasticization molding technologies were classified into three types—ultrasonic pressing (UP), ultrasonic plasticizing and pressing (UPP), and ultrasonic plasticization injection molding (UPIM). The effects of these configurations on melt fluidity and filling performance were evaluated and compared through slit flow tests. The interaction mechanisms between polymer melts and templates were elucidated based on melt pressure measurements and morphological changes in nickel micropillar arrays and silicon templates after molding. The results indicated that polymer melt exhibits improved flow behavior within microcavities when under the coverage area of the ultrasonic sonotrode action surface and subjected to the axial ultrasonic vibration. Continuous ultrasonic vibration contributed to sustaining melt fluidity during micropore filling. Among the three technologies, the most complex and intense mechanical interactions on the template microstructure were observed in UP, followed by UPP and then UPIM.

## 1. Introduction

The rapid development of micro-electromechanical systems (MEMS), biomedicine, and micro-optics has increased the demand for polymer microstructures (microcomponents) with diverse material properties, ease of processing, and high molding efficiency [1,2]. However, conventional thermoplastic molding technologies face several challenges. Incomplete filling is common in micron-to submicron-scale cavities [3,4]. Moreover, the high-temperature and high-pressure conditions often cause polymer degradation, adversely affecting the mechanical and optical properties of the final products. The plasticization process, driven by screw-induced shear and thermal energy, is also energy-intensive and results in extended cycle times. To address these challenges, studies have incorporated ultrasonic plasticization into polymer micromolding to leverage its unique energy conversion mechanisms [5,6,7]. Ultrasonic plasticization achieves rapid plasticization of thermoplastic polymers by employing high-frequency mechanical vibrations to generate thermal effects [8,9,10], cavitation [11], and mechanical effects [12,13]. It simultaneously reduces melt viscosity and improves the filling efficiency of micropores. Compared with conventional methods, ultrasonic plasticization molding offers several advantages, including improved microstructure formation, higher material utilization, lower energy consumption, and shorter cycle times [14,15,16].

Various micromolding technologies employ ultrasonic vibration as the energy source for plasticizing thermoplastics, including room-temperature ultrasonic embossing/imprinting (UE/I) [17,18,19], ultrasonic powder molding (UPM) [20,21,22], and ultrasonic plasticization and injection molding (UPIM) [23,24,25], along with other derived techniques [26,27]. In both UE/I and UPM, plasticization of the raw material, melt flow, and microcavity filling occur entirely within the coverage area of the ultrasonic sonotrode’s action surface. Thus, the entire molding process is subjected to axial ultrasonic vibration, which is the dominant vibration mode. By contrast, in UPIM, only the plasticization step takes place under the sonotrode. Once the melt enters the sprue channel, it is no longer influenced by axial ultrasonic vibration. Whether this difference affects the fluidity and filling behavior of polymer melts under microscale conditions remains unverified. This gap limits the advancement of ultrasonic molding technologies, the development of novel processes, and the rational selection of suitable techniques for fabricating polymer microstructures. Furthermore, in UE/I and UPM, the raw material is positioned between the ultrasonic sonotrode and the microcavity. As a result, plasticization and cavity filling occur concurrently, leading to more complex flow dynamics and stronger interactions between the polymer melt and the template microstructure compared to UPIM. However, these effects have not been systematically investigated, thereby restricting the development and broader adoption of ultrasonic molding techniques.

**Figure 1:**
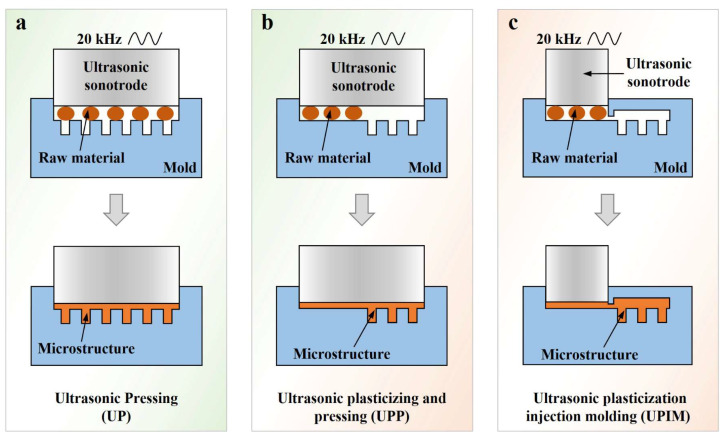
Schematic diagrams of the UP (**a**), UPP (**b**), and UPIM (**c**) processes.

Based on the positioning of raw material during plasticization and whether the ultrasonic sonotrode action surface encompasses the microcavity, this study categorized existing ultrasonic plasticization molding technologies for polymers into three types: (1) ultrasonic pressing (UP), (2) ultrasonic plasticizing and pressing (UPP), and (3) UPIM. As shown in Figure 1a, in UP, the raw material is placed directly above the microcavity, and both the material and cavity lie within the sonotrode’s action area. Plasticization and cavity filling occur simultaneously. UE/I, UPM, and related derivative processes fall under this category. Proposed in a prior study [28], UPP positions the raw material away from the microcavity, although both remain under the influence of the sonotrode (Figure 1b). In UPP, material plasticization is followed by microcavity filling. As illustrated in Figure 1c, in UPIM, the raw material is positioned under the sonotrode, away from the microcavity. Plasticization occurs in a barrel, and the melt subsequently flows through a sprue channel and fills the microcavity.

A mold incorporating rectangular slits was developed to characterize melt flow behavior in the absence of commercial rheometers capable of accurately evaluating the rheology of polymer melts plasticized by ultrasonic vibration. The effects of the differences among UP, UPP, and UPIM on melt fluidity, filling capability, and interactions with the template microstructure were examined through slit flow tests. Given that microstructure formation in UP involves concurrent plasticization and cavity filling, the effects of process parameters on melt behavior and melt–template interaction were further investigated for this process. Comparative analysis of the three methods provides improvement directions or solution ideas for incomplete filling in micro- and nanostructure fabrication. It also offers essential theoretical and practical guidance for selecting optimal polymer microstructure molding techniques. In addition, analysis of the UP process provides valuable insights into the complex rheological behavior of polymer melts during simultaneous plasticization and flow, supporting improved template design, material selection, and broader implementation of UP-based approaches.

## 2. Experimentation

### 2.1. Materials

Three types of raw materials were used in the study: polypropylene (PP) pellets (approximately 3 × 2.5 × 5 mm^3^) supplied by Korea Chungnam Lotte Chemical Co. (Seoul, Republic of Korea); polystyrene (PS) pellets (approximately 3 × 4 × 3 mm^3^) from Shandong UsoIf Chemical Technology Co. (Linyi, China); and polycarbonate (PC) pellets (approximately 2.5 × 3 × 3 mm^3^) from Ningbo Dafeng Jiangning New Material Technology Co. (Ningbo, China). PP is a semi-crystalline polymer with a melting point of approximately 160 °C. PS and PC are amorphous polymers with glass transition temperatures of approximately 100 °C and 150 °C, respectively.

Nickel plates (thickness: 8 mm) supplied by Qinghe County Boguan Metal Materials Co. (Xingtai, China) were used to fabricate nickel micropillar arrays. Single-sided polished silicon wafers (thickness: 500 μm) supplied by Jing Yan Electronic Technology Co. (Quzhou, China) were used to produce silicon templates.

### 2.2. Equipment and Mold

**Figure 2:**
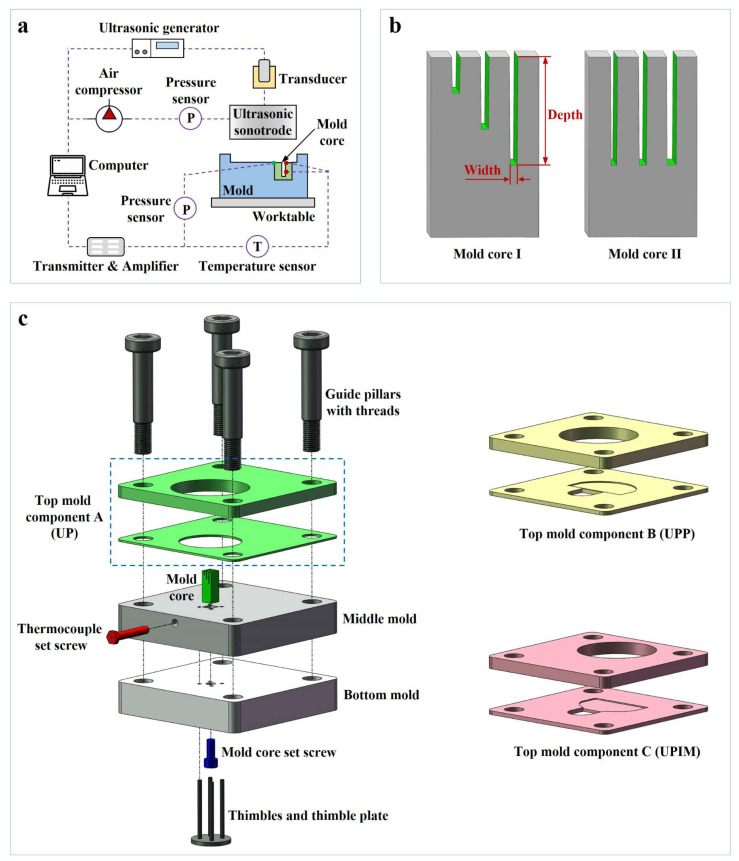
Equipment components and mold design. (**a**) Equipment components. (**b**) Mold core design. (**c**) Mold design.

As shown in Figure 2a, the ultrasonic loading system used in the study comprised an ultrasonic generator, a transducer, an ultrasonic sonotrode, a worktable, an air compressor, and a pressure sensor. The system operated at a resonant frequency of 20 kHz with a maximum amplitude of 60 μm. Two temperature sensors were employed to measure the polymer melt temperature at the inlet and within the microcavity, while a pressure sensor monitored melt pressure at the microcavity inlet.

**Figure 3:**
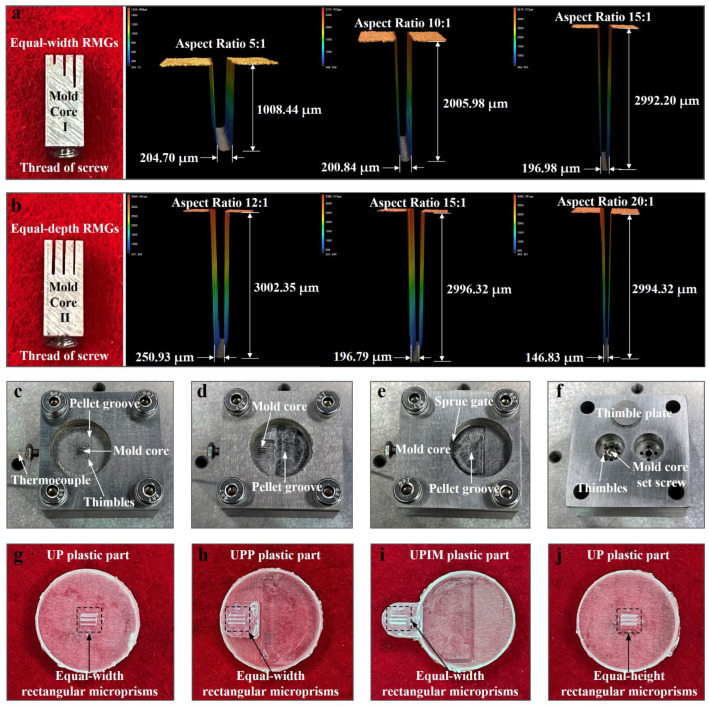
Photographs of the mold cores, molds, and molded parts. (**a**) Mold core I and the morphology and dimensions of its microgrooves. (**b**) Mold core II and the morphology and dimensions of its microgrooves. (**c**–**e**) Assembled molds for UP, UPP, and UPIM, respectively. (**f**) Bottom view of the assembled mold. (**g**–**i**) Plastic parts formed by UP, UPP, and UPIM using mold core I. (**j**) Plastic part formed by UP using mold core II.

To investigate the effect of microcavity size on polymer melt fluidity at the microscale, two 316 stainless steel mold cores with dimensions 3 mm × 3 mm × 8 mm were designed: mold core I and mold core II. As illustrated in Figure 2b, mold core I contained three rectangular microgrooves of equal width and varying depth (equal-width RMGs), whereas mold core II featured three microgrooves of equal depth and different widths (equal-depth RMGs). The microgrooves were fabricated using low-speed wire electrical discharge machining (LS-WEDM). A 1° pull-out slope was added to each microgroove to facilitate demolding. The machined cores and the morphology and dimensions of the microgrooves are shown in Figure 3a,b.

As shown in Figure 2c, the mold assembly encompassed a bottom mold, middle mold, top mold components, mold core, thimbles, and a thimble plate. The assembly was centered and fixed on the worktable using guide pillars. Interchangeable mold components A, B, and C were designed for the UP, UPP, and UPIM processes, respectively, facilitating adaptation to all three molding modes. To eliminate variability from the mold designs, the plasticization-to-core distance in components B and C was standardized, and the mold cavity volume formed by the ultrasonic sonotrode, trough, and mold wall was kept consistent across all configurations. A thermocouple set screw was embedded in the middle mold to enable temperature measurement at the inlet and within the microcavity.

Figure 3c–e display the assembled molds used for UP, UPP, and UPIM, respectively, and Figure 3f presents the bottom view of the mold assembly. The plastic parts fabricated using mold core I (equal-width rectangular microgrooves, RMGs) through UP, UPP, and UPIM are shown in Figure 3g–i, respectively. Figure 3j presents the plastic part formed by UP using mold core II (equal-depth RMGs). All plastic parts featured rectangular microprisms corresponding to the mold microgrooves, confirming the accuracy of both the mold and mold core designs. The successful replication of these structures validated the suitability of the mold system for comparative analyses of the UP, UPP, and UPIM processes.

### 2.3. Methodology

The effects of process parameters on polymer melt fluidity in the UP, UPP, and UPIM processes were evaluated through single-factor experiments. The investigated parameters included ultrasonic action time (UA time), ultrasonic amplitude, and ultrasonic loading pressure. Specific values are provided in Table 1. The ultrasonic trigger force and holding time were fixed at 500 N and 8 s, respectively. For each experimental condition, the height of the formed rectangular microprisms was measured in five samples, and the arithmetic mean was used for analysis. To investigate the effect of microgroove depth on melt fluidity, three rectangular microgroove designs with identical widths but different depths were used. Similarly, to examine the influence of microgroove width, three designs with identical depths but varying widths were employed. The cross-sectional dimensions of these microgrooves are listed in Table 2, where “Actual Dimension” refers to the measured dimensions of the microgrooves obtained through LS-WEDM.

Melt temperatures at the inlet of the microgroove and 2 mm downstream, along with inlet melt pressures, were measured to identify the factors contributing to variations in melt fluidity and microstructure damage in UP with increasing ultrasonic action (UA) time, as well as to assess differences among UP, UPP, and UPIM under identical process conditions. For these experiments, the ultrasonic amplitude and loading pressure were fixed at 60 µm and 300 kPa, respectively.

A nickel micropillar array was employed as a template to investigate microstructure damage in UP with increasing UA time and to compare it with UPP and UPIM. Micropillars with a design size of 100 × 100 × 200 µm^3^ were fabricated through LS-WEDM on a 3 × 3 × 8 mm^3^ nickel mold core (Figure S1). Additionally, single-side polished silicon wafers (2.8 × 2.8 × 0.5 mm^3^) were used as alternative templates to enable further comparative evaluation of damage across the three processes. Each silicon template was reused three times to ensure experimental rigor before analysis.

### 2.4. Characterization

The morphologies of the microgrooves, nickel micropillar arrays, and silicon wafers, both before and after use, were examined using a laser scanning confocal microscope (LSCM; VK-250, Keyence, Osaka, Japan). The heights of the formed rectangular microprisms were measured using an optical microscope (JT-H300, Shenzhen Jintuo Youcheng Technology, Shenzhen, China).

X-ray diffraction (XRD; MiniFlex600, Rigaku, Japan) was conducted over a 2θ range of 10–100° to evaluate changes in crystal structure during microstructure formation in UP, UPP, and UPIM. Differential scanning calorimetry (DSC; DSC8000, PerkinElmer, Waltham, MA, USA) was used to determine the melting point and enthalpy of the formed rectangular microprisms. Samples were heated under a nitrogen atmosphere from 25 to 200 °C at a rate of 10 °C/min. Crystallinity (*X_c_*) was calculated using Equation (1) [29]:

(1) $$X_{c}=\frac{\nabla H_{m}}{\nabla H_{m}^{0}}\times 100\%,$$

where $\nabla H_{m}$ is the measured melting enthalpy, and $\nabla H_{m}^{0}$ is the melting enthalpy of fully crystalline PP (177 J/g) [29]. Fourier transform infrared (FTIR) spectroscopy was performed on both the PP pellets and the formed rectangular microprisms over the spectral range of 400–4000 cm^−1^ using an FTIR spectrometer (Nicolet 6700, Nicolet, Madison, WI, USA). The hardness and elastic modulus of the rectangular microprisms were measured using a nanoindenter (DSI; T1950, Hysitron, Eden Prairie, MN, USA) equipped with a conical triangular tip. Microprisms with an aspect ratio of 15 were selected for testing. Under each experimental condition, three samples were measured, with nine indentation points designated per sample (Figure S2). Outlier data were excluded, and the arithmetic mean of the remaining values was used as the representative value for each sample.

## 3. Results and Discussion

### 3.1. Characterization and Comparison of the Fluidity

To accurately assess the fluidity of the ultrasonic plasticized polymer melt, the testing method must replicate the ultrasonic plasticization molding process. Ultrasonic vibration-induced plasticization proceeds via an inside-out heating mechanism [30]. Therefore, conventional rheological instruments—such as rotary rheometers, high-pressure capillary rheometers, and melt flow index (MFI) testers—are unsuitable because of their reliance on outside-in heat conduction. These instruments cannot effectively measure or characterize melt fluidity under ultrasonic conditions. To address this limitation, a slit flow test based on rectangular microgrooves was adapted for UP, UPP, and UPIM. Fluidity was quantified based on the height of the formed rectangular microprisms.

**Figure 4:**
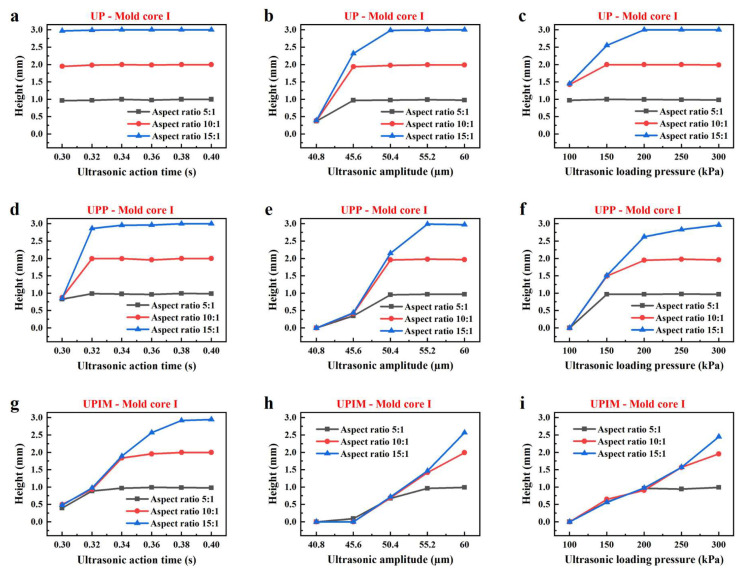
Influence of process parameters on the fluidity of PP melt in mold core I. Height of rectangular microprisms formed by UP versus UA time (**a**), amplitude (**b**), and loading pressure (**c**). Height of rectangular microprisms formed by UPP versus UA time (**d**), amplitude (**e**), and loading pressure (**f**). Height of rectangular microprisms formed by UPIM versus UA time (**g**), amplitude (**h**), and loading pressure (**i**).

**Figure 5:**
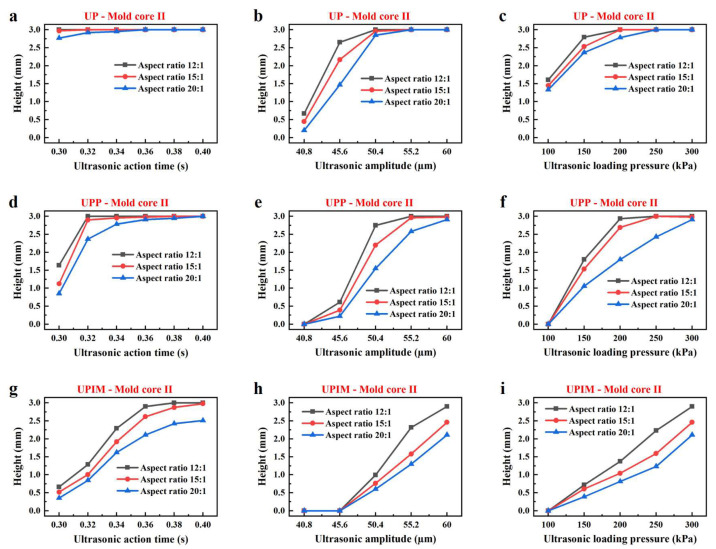
Influence of process parameters on the fluidity of PP melt in mold core II. Height of rectangular microprisms formed by UP versus UA time (**a**), amplitude (**b**), and loading pressure (**c**). Height of rectangular microprisms formed by UPP versus UA time (**d**), amplitude (**e**), and loading pressure (**f**). Height of rectangular microprisms formed by UPIM versus UA time (**g**), amplitude (**h**), and loading pressure (**i**).

As shown in Figure 4, the height of the PP rectangular microprisms increased with UA time, ultrasonic amplitude, and loading pressure across all three processes. Figure 5 shows the same trend. This indicates that increasing UA time, amplitude, and loading pressure enhanced the fluidity of the PP melt, thereby improving its ability to fill microcavities. As indicated in Figure 4, for each forming, when the microgrooves were not fully filled, the heights of the three resulting microprisms were nearly identical. However, Figure 5 reveals a distinct difference. Increased microgroove width correlated with greater microprism height. This indicates that melt fluidity was more strongly influenced by width than by depth, and wider microgrooves promoted improved fluidity of the PP melt. In addition, these results also demonstrate that, in all three processes, the effects of process parameters and microcavity dimensions on melt fluidity followed a consistent trend across UP, UPP, and UPIM.

**Figure 6:**
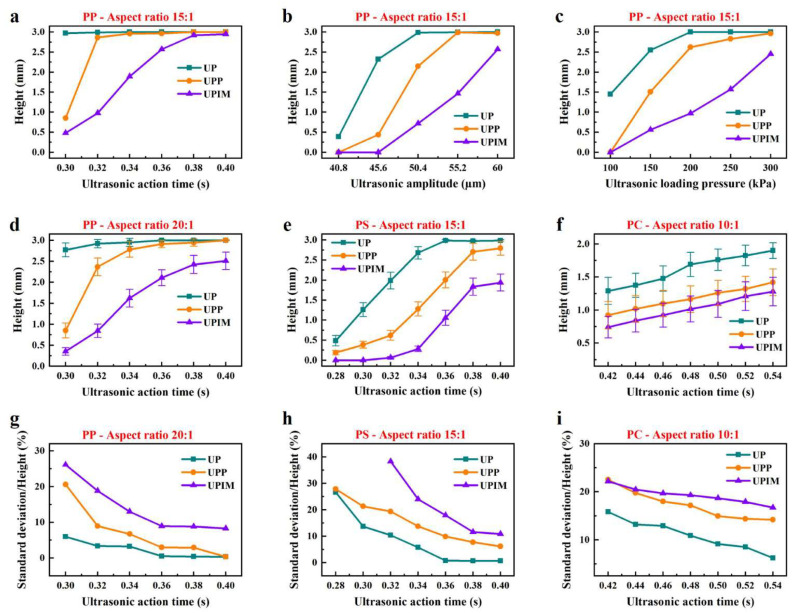
Comparison of polymer melt fluidity and process stability in UP, UPP, and UPIM. Height of PP microprisms formed in the microgroove (AR = 15:1) versus UA time (**a**), amplitude (**b**), and loading pressure (**c**). (**d**) Height of PP microprisms formed in the microgroove (AR = 20:1) versus UA time. Height of rectangular microprisms versus UA time for PS (**e**) and PC (**f**). Ratio of standard deviation to arithmetic mean of microprism heights for five replicates of PP (**g**), PS (**h**), and PC (**i**). Error bars represent the standard deviation of microprism height.

The experimental data from Figure 4 and Figure 5 were extracted and replotted as shown in Figure 6a–d for a direct comparison of polymer melt fluidity during micropore filling in UP, UPP, and UPIM. Under identical process parameters, the height of PP microprisms followed the order UP > UPP > UPIM. As shown in Figure 6d, UP produced microprisms with an aspect ratio (AR) of 20:1 at a UA time of 0.36 s, while UPP required 0.40 s to achieve the same AR. By contrast, UPIM produced microprisms with a height of approximately 2.51 mm at 0.40 s, corresponding to an AR of 16.7. Based on the trend in microprism height with increasing UA time, UPIM is difficult to achieve an AR of 20:1 under an ultrasonic amplitude of 60 μm and loading pressure of 300 kPa. These results indicate that PP melt exhibits the highest fluidity in UP, followed by UPP and UPIM. Additionally, UP is more effective in fabricating high-AR microstructures. This trend was further validated through additional tests using PS and PC as raw materials. As shown in Figure 6e,f, the same pattern was observed: UP produced the tallest microprisms, followed by UPP and UPIM. These findings confirm that UP outperforms UPP and UPIM in both melt fluidity and microstructure formation.

To assess process stability, standard deviations of the heights of five replicates were plotted as error bars in Figure 6d–f. For PP, the standard deviation ranged from 0.010 to 0.165 mm in UP, 0.011–0.212 mm in UPP, and 0.091–0.215 mm in UPIM. For PS, the corresponding ranges were 0.020–0.206 mm for UP, 0.054–0.210 mm for UPP, and 0.024–0.213 mm for UPIM. For PC, the ranges were 0.118–0.204 mm for UP, 0.188–0.208 mm for UPP, and 0.163–0.216 mm for UPIM. However, these standard deviation ranges did not clearly distinguish process stability among the three methods. Therefore, the ratio of the standard deviation to the arithmetic mean of the sample heights shown in Figure 6d–f was calculated, and the results are shown in Figure 6g–i. Based on this normalized metric, UP demonstrated the highest process stability, followed by UPP and then UPIM. Across all three processes, process stability improved with increasing UA time.

### 3.2. Melt Temperature and Pressure

Melt temperature and pressure critically influence polymer melt fluidity; higher values generally enhance flow behavior [31,32]. To evaluate the flow characteristics of polymer melt plasticized by ultrasonic vibration, both temperature and pressure were measured.

**Figure 7:**
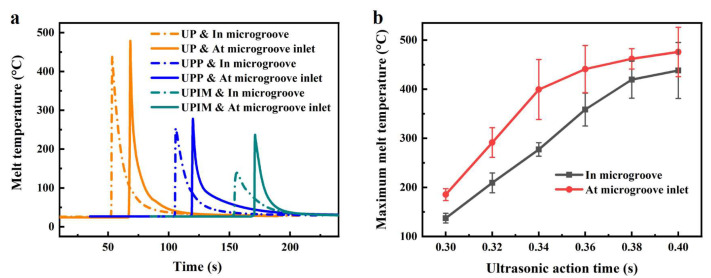
(**a**) Temperature curves of PP melt at the inlet and inside the microgroove in UP, UPP, and UPIM under a UA time of 0.40 s. (**b**) Maximum melt temperatures at the inlet and inside the microgroove in UP as a function of UA time. Error bars represent standard deviations of the corresponding measurements.

Figure 7a presents the measured temperature profiles of PP melt at the inlet and inside the microgroove for UP, UPP, and UPIM. The temperature increased rapidly and then decreased sharply. Under identical UA times, the maximum melt temperatures at both locations, which are the peak values of the temperature profile, were highest in UP, followed by UPP, and lowest in UPIM. The melt continuously dissipated heat during flow and filling of the microgroove, resulting in a higher melt temperature at the inlet than inside the microgroove. Table 3 summarizes the maximum melt temperatures at the inlet and inside the microgroove, along with the difference between them. Although the melt in UPIM exhibited lowest absolute temperatures, it exhibited the largest temperature difference (97.9 °C), indicating a more pronounced temperature drop during micropore filling. This indicates that the limited coverage of the ultrasonic sonotrode in UPIM reduces the ability to retain melt temperature compared to UP and UPP.

As illustrated in Figure 7b, in UP, the maximum temperatures at the inlet and inside the microgroove increased with UA time, though the rate of increase diminished progressively. Longer UA times induced greater conversion of ultrasonic vibration energy into heat due to friction and viscoelastic effects of the PP pellets. However, these effects weakened as plasticization advanced, leading to reduced heat generation and slower temperature increase. The temperature difference initially increased with UA time but began to decline beyond 0.34 s, indicating improved melt temperature retention during micropore filling under prolonged ultrasonic vibration.

**Figure 8:**
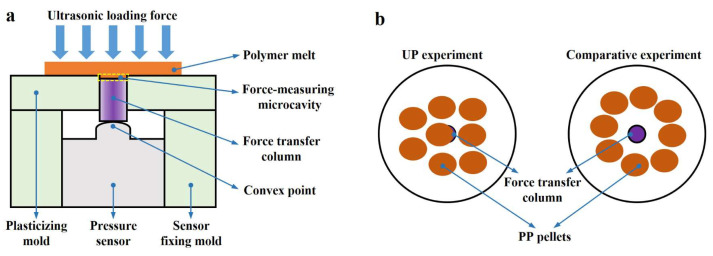
(**a**) Schematic diagram of the melt pressure measurement setup for UP, UPP, and UPIM. (**b**) Two placement configurations for PP pellets in UP: on the left, pellets were positioned directly above the force transfer column (UP experiment), while on the right, they were placed around the force transfer column (comparative experiment).

A dedicated experimental setup was developed to measure melt pressure during ultrasonic vibration plasticization and subsequent melt flow, as shown in Figure 8a. In this setup, the pressure of the polymer melt was transmitted to a pressure sensor via a 2 mm diameter force transfer column. The column was positioned at the same location as the mold core to ensure accurate and efficient transmission, with its upper surface set 0.2 mm lower than that of the plasticizing mold. The resulting height difference formed a microcavity, referred to as the force-measuring microcavity.

**Figure 9:**
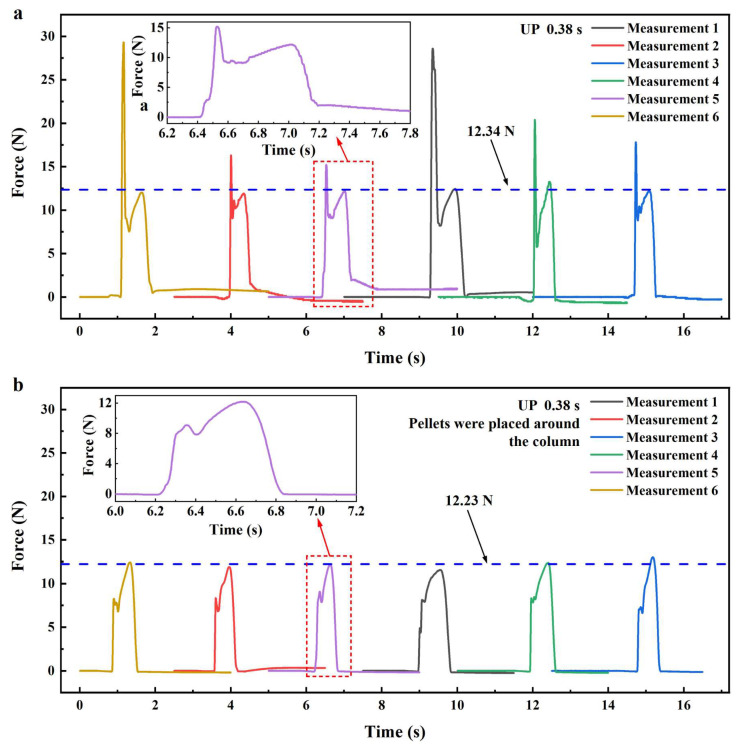
Force curves obtained by the pressure sensor in UP at a UA time of 0.38 s: (**a**) PP pellets placed directly above the force transfer column; (**b**) PP pellets placed around the force transfer column.

In UP, when the UA time was set to 0.38 s, the force curves obtained using the setup in Figure 8a are shown in Figure 9a. The force curve exhibited two distinct peaks, with the first peak significantly larger than the second. Within approximately 0.2 s, the force rose sharply before declining rapidly, followed by a second rise and fall over 0.5–0.7 s. Among the six measurements, the first peak exhibited substantial fluctuations without a clear pattern, while the second peak remained relatively stable, centering around 12.34 N (the arithmetic mean). In UP, PP pellets were placed directly above the microcavities. To improve measurement accuracy, the pellets were positioned randomly near the center of the plasticizing mold’s upper surface, resulting at least one pellet was located directly above the force transfer column, as shown in the left inset of Figure 8b. Based on the timing and duration of the two peaks, the results in Figure 9a can be interpreted as follows. The first peak was due to rapid filling of the force-measuring microcavity by partially plasticized PP pellets. Before full plasticization, the force transfer column experienced a “hard” pressure transmitted from the sonotrode through the unplasticized portion of the pellets, resulting in a sharp increase and a prominent peak in the force curve. As plasticization proceeded and the pellets fully melted, the force rapidly declined. The magnitude of the initial pressure varied with pellet size and placement, accounting for the large fluctuations in the first peak. Once plasticization was complete, the ultrasonic sonotrode continued to apply pressure to the melt, resulting in a second, more stable peak. This second peak diminished as the melt cooled and solidified and was largely unaffected by pellet placement, resulting in minimal variation across measurements.

Comparative experiments were performed under identical process conditions to verify the presence of “hard” pressure and validate the preceding analysis. In these trials, PP pellets were placed around but not directly above the force transfer column, as shown in the right diagram of Figure 8b. The resulting force curves in Figure 9b show a marked reduction in the first peak, which also became more consistent across the six measurements. This first peak is thus attributed to the melt filling the force-measuring microcavity after complete plasticization, rather than partial melting. The contrast in magnitude and variability of the first peak between UP and the comparative experiment confirms the presence of “hard” pressure in UP. The second peak in Figure 9b closely matches that in Figure 9a, with an average value of 12.23 N, nearly identical to the 12.34 N observed in UP, further supporting its origin from sonotrode pressure on the fully plasticized melt.

**Figure 10:**
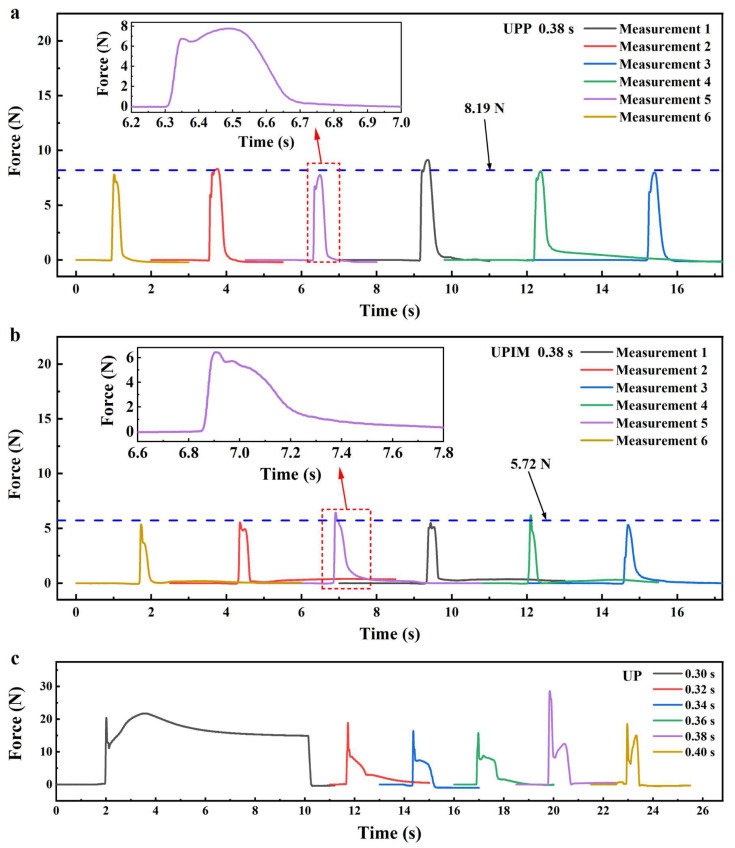
Force curves based on the measurements of the pressure sensor in UP, UPP, and UPIM. (**a**) Force curves in UPP at a UA time of 0.38 s. (**b**) Force curves in UPIM at a UA time of 0.38 s. (**c**) Force curves in UP at UA times of 0.30, 0.32, 0.34, 0.36, 0.38, and 0.40 s, respectively.

Force curves in UPP under the same process parameters are shown in Figure 10a. Although the overall trend resembles that of UP, the first peak is significantly smaller than the second and lacks the magnitude and fluctuation observed in UP, indicating the absence of “hard” pressure in UPP. The second peak averages at 8.19 N, and the total duration of the two peaks ranges from 0.3 to 0.5 s, both lower than in UP. By contrast, the force curve for UPIM in Figure 10b shows a single peak with an average value of 5.72 N and a duration of 0.3–0.4 s, both lower than those in UP and UPP. These results demonstrate that melt pressure during micropore filling is highest and most complex in UP, followed by UPP, and lowest in UPIM. The duration of melt pressure follows the same order.

Force curves in UP at various UA times are shown in Figure 10c. As UA time increases, significant changes appear, primarily after the first peak. At shorter UA times (e.g., 0.30 s), a sustained high force, occasionally exceeding the first peak, follows the initial rise and persists through the pressure-holding phase. At intermediate times (e.g., 0.34 s), the curve no longer rises after the first peak but gradually declines, and the second peak disappears. With further increases in UA time (e.g., 0.38 s), the force curve rises again after the first peak, forming a distinct second peak. The longer the UA time, the greater the magnitude of this second peak. Despite variations in the force curves, the overall trend indicates a gradual increase in melt pressure with longer UA durations. These results confirm that extended ultrasonic vibration elevates and maintains melt pressure more effectively.

The measured melt temperature and pressure account for the enhanced microstructural formation and improved melt fluidity observed in UP relative to UPP, and for the superior performance of UPP compared to UPIM. These results also elucidate the mechanism by which increased UA time in UP promotes more effective micropore filling and further enhances melt flow behavior. From a process standpoint, in UPIM, the polymer melt exits the effective range of the ultrasonic sonotrode’s active surface immediately upon entering the sprue channel. By contrast, in both UP and UPP, the melt remains within the sonotrode’s influence throughout the micropore-filling stage, directly subjected to axial ultrasonic vibration. Additionally, in the designed forming mold, the distance traveled by the melt from the plasticization position to the microgroove in UPP was equal to that in UPIM. These, combined with the experimental results, confirm that axial ultrasonic vibration—the predominant vibration mode—acts on the polymer melt to improve its fluidity during micropore filling and to sustain the enhanced flow characteristics. In UP, continuous ultrasonic excitation further contributes to maintaining optimal melt fluidity throughout the entire filling process. During micropore filling, axial ultrasonic vibration directly acted on the melt, which not only helped maintain the polymer’s viscoelastic heat generation effect but also fully leveraged the stirring action of ultrasonic vibration, enabling the melt to more easily penetrate the micro- and nanoscale pores. While in UPIM, once the melt entered the sprue channel, it was no longer directly subjected to axial ultrasonic vibration. Consequently, ultrasonic vibration could not fully exert the aforementioned effects.

### 3.3. Ultrasonic Plasticization and Melt Flow Behavior in up

**Figure 11:**
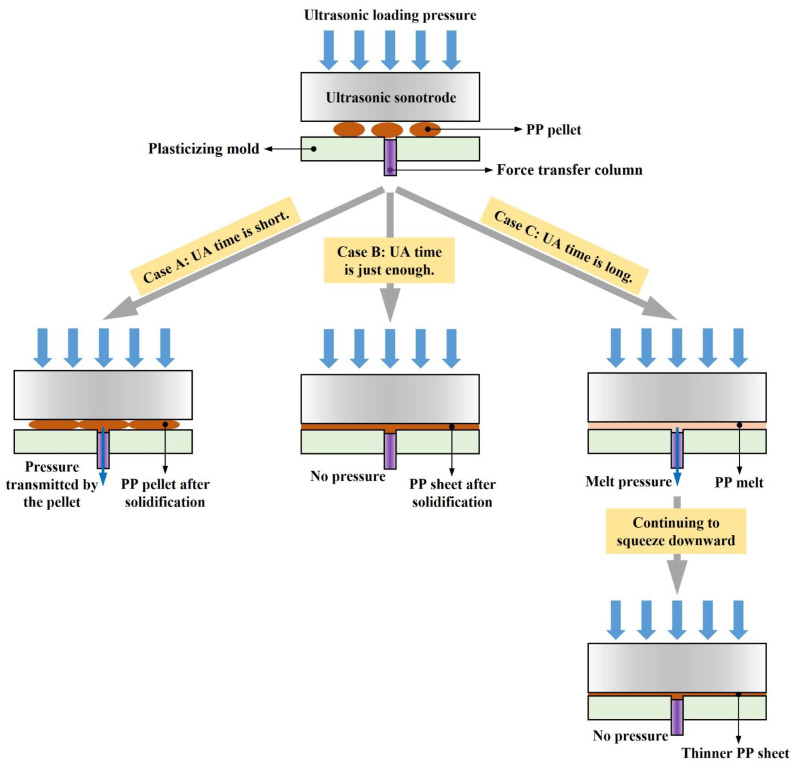
Schematic diagrams of ultrasonic plasticization and melt flow behavior in UP as UA time increases.

The variation in force curve trends with increasing UA time reflects changes in ultrasonic plasticization and melt flow behavior in UP. These behaviors are illustrated in Figure 11, which contextualizes the differences observed after the first peak in the force curves by presenting the corresponding plasticization and flow mechanisms at different UA times.

When UA time is short (Case A), the energy delivered by ultrasonic vibration is insufficient to fully plasticize and fuse all polymer pellets into a continuous melt. The pellet positioned above the force transfer column undergoes only partial plasticization, filling the force-measuring microcavity while sharing the loading pressure with other incompletely plasticized pellets. Consequently, part of the pressure is transmitted to the transfer column, allowing the second peak in the force curve to persist throughout the pressure-holding phase.

At a critical UA time (Case B), the ultrasonic energy becomes sufficient to completely plasticize the pellets, enabling the melt to flow and fuse into a homogeneous structure. The fully plasticized melt fills the microcavity and spreads uniformly across the upper surface of the plasticizing mold, where it undergoes immediate cooling and solidification. Once solidified, the melt resists further compression from the ultrasonic sonotrode, causing the measured force to decline after the first peak and resulting in the disappearance of the second peak. During the pressure-holding stage, the solidified sheet absorbs the sonotrode’s pressure and transfers it to the mold, ultimately reducing the measured force to 0 N.

With prolonged UA time (Case C), the polymer melt remains in a molten state for an extended duration under continuous ultrasonic vibration. This sustained molten state permits ongoing downward compression by the ultrasonic sonotrode, causing the melt to be extruded outward through the gap between the mold and the ultrasonic sonotrode. As flow resistance increases, the squeezing force intensifies, resulting in a secondary rise in the force curve and the emergence of a second peak. A longer UA time produces deeper compression and a more pronounced second peak.

### 3.4. Template Microstructure Damage Analysis and Comparison

Damage to the template microstructure induced by polymer melt during microcavity filling has a critical influence on formability, process scalability, and manufacturing cost. In the fabrication of polymer micropore arrays with high ARs, the template, typically a high-AR micropillar array, is susceptible to deformation or bending because of the stamping effect exerted by the polymer melt during flow. To investigate and compare the extent of template damage across different processing methods, nickel micropillar arrays were fabricated and employed as templates in UP, UPP, and UPIM.

**Figure 12:**
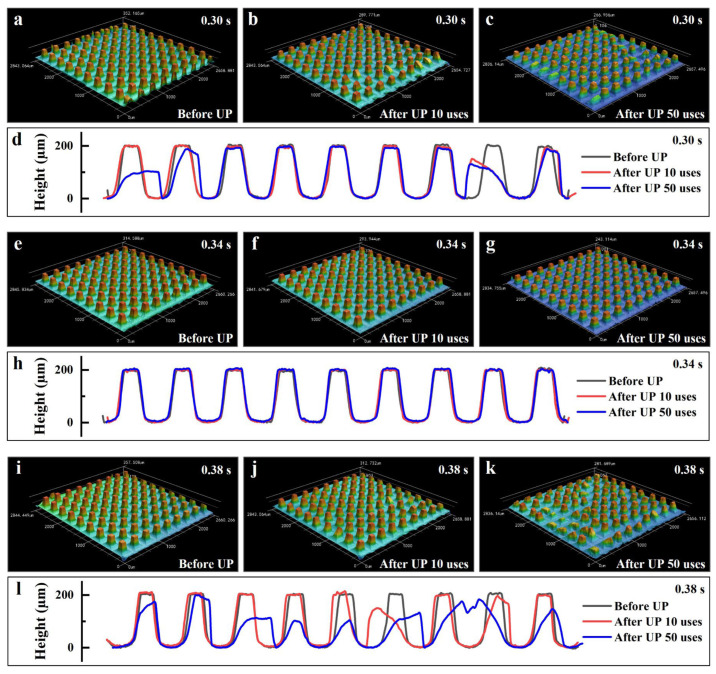
Influence of UA time on the damage of the template microstructure in UP. Morphologies of the nickel micropillar arrays before (**a**), after 10 (**b**), and 50 (**c**) uses in UP at a UA time of 0.30 s. (**d**) Contours of a specific column of micropillars showing greater deformation in (**a**), (**b**), and (**c**), respectively. Before (**e**), after 10 (**f**), and 50 (**g**) uses in UP at a UA time of 0.34 s. (**h**) Contours of a specific column of micropillars showing greater deformation in (**e**), (**f**), and (**g**), respectively. Before (**i**), after 10 (**j**), and 50 (**k**) uses in UP at a UA time of 0.38 s. (**l**) Contours of a specific column of micropillars showing greater deformation in (**i**), (**j**), and (**k**), respectively.

The morphologies of the nickel micropillar arrays before and after use in UP at UA times of 0.30, 0.34, and 0.38 s are presented in Figure 12. Corresponding micropore array morphologies (replicated plastic parts) are presented in Figure S3. As illustrated in Figure 12a–c, significant deformation and bending of micropillars occurred at a UA time of 0.30 s, even after only 10 uses. This deformation became more severe and widespread following 50 uses. The progression of deformation is further visualized through micropillar contour comparisons in Figure 12d. By contrast, at a UA time of 0.34 s (Figure 12e–h), no substantial deformation was observed even after 50 cycles, and the micropillar geometry remained largely unchanged. However, when the UA time was extended to 0.38 s (Figure 12i–l), extensive bending deformation was evident in most micropillars after repeated use. These results indicate that micropillar deformation in UP does not scale linearly with increasing UA time. A comparison with the force curves at different UA times (Figure 10c) reveals that deformation was not associated with the initial force peak, which corresponds to the plasticization stage of the PP pellets. This observation suggests that the nickel micropillar arrays possess sufficient mechanical strength to withstand ultrasonic plasticization without structural failure. Rather, the differences in deformation are attributed to variations in the post-peak force behavior, including both the force magnitude and the trend following the first peak. These factors directly influence the extent of micropillar damage.

**Figure 13:**
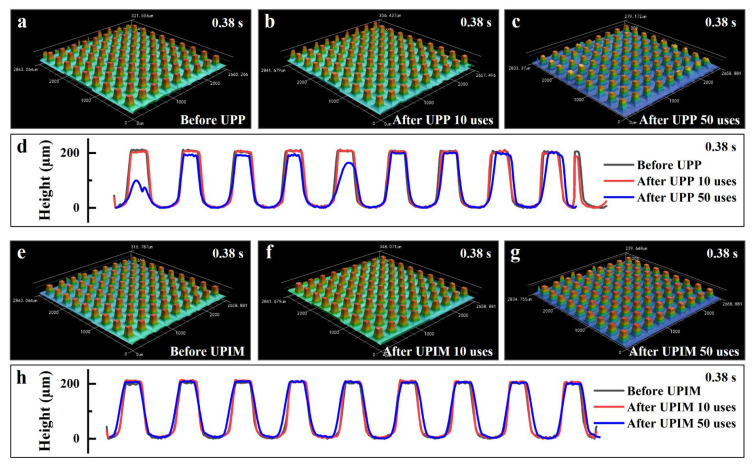
Comparison of template microstructure damage among UP, UPP and UPIM. Morphologies of the nickel micropillar arrays before (**a**), after 10 (**b**) and 50 (**c**) uses in UPP at a UA time of 0.38 s. (**d**) Contours of a particular column of micropillars with larger deformations in (**a**), (**b**), and (**c**), respectively. Before (**e**), after 10 (**f**) and 50 (**g**) uses in UPIM at a UA time of 0.38 s. (**h**) Contours of a particular column of micropillars with larger deformations in (**e**), (**f**), and (**g**), respectively.

The morphologies of the nickel micropillar arrays before and after use in UPP and UPIM at a UA time of 0.38 s are shown in Figure 13. The corresponding micropore array morphologies (replicated plastic parts) are presented in Figure S4. As illustrated in Figure 13a–d, no significant deformation occurred in UPP after 10 uses, although slight deformation was observed in some micropillars after 50 uses. By contrast, Figure 13e–h show that the micropillars in UPIM remained structurally intact even after 50 uses, exhibiting no detectable deformation. These results indicate that, under identical process conditions, micropillar deformation was most severe in UP, followed by UPP, while UPIM caused the least structural damage.

**Figure 14:**
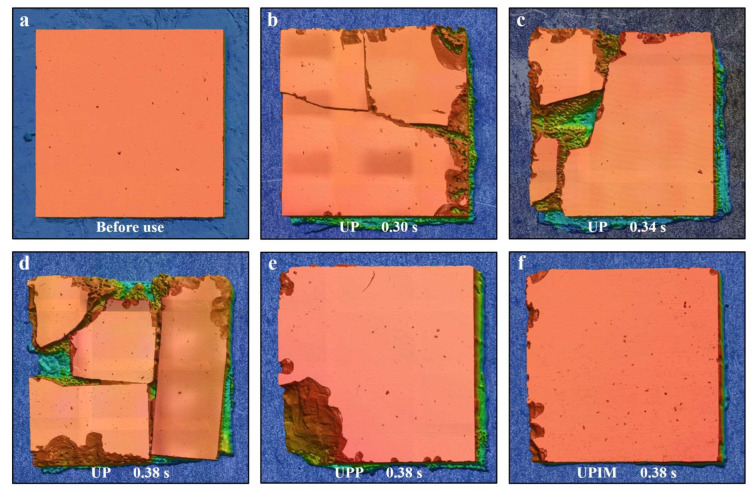
Damage to silicon templates in UP, UPP, and UPIM. (**a**) Silicon template before use. (**b**), (**c**), and (**d**) Silicon templates after use in UP at UA times of 0.30, 0.34, and 0.38 s, respectively. (**e**) and (**f**) Silicon templates after use in UPP and UPIM, respectively, at a UA time of 0.38 s.

To further assess template damage in UP, UPP, and UPIM, fragile silicon wafers were also used as templates. Their morphologies before and after use are shown in Figure 14. In UP, the silicon templates exhibited severe fracturing at UA times of 0.30 s, 0.34 s, and 0.38 s, with fracture severity increasing with UA time. Notably, the damage evolution in silicon templates differed from that in nickel micropillars. Because of the brittle nature of silicon, fracturing likely occurred during the initial stage of ultrasonic plasticization of the PP pellets. The resulting fragments subsequently collided under intense vibrations, causing further breakage and increased damage at longer UA times. By contrast, the silicon templates in UPP and UPIM remained intact, exhibiting only localized notching at the edges and corners. The degree of notching was more pronounced in UPP than in UPIM. These observations indicate not only differences in the extent of damage but also distinct damage mechanisms across the three processes. UP led to extensive fracturing, while UPP and UPIM caused only edge and corner chipping, with the central region remaining structurally intact. Considering process differences, it can be deduced that edge notching was caused by collisions between the silicon template and mold induced by melt squeezing in the flow direction.

The combined of nickel micropillar deformation and silicon template fracture fracturing confirms that UP caused the most severe template damage, followed by UPP, while UPIM resulted in the least. Moreover, in UP, the damage mechanism depends on the structural strength of the template. According to the experimental results and the aforementioned analysis, the nickel micropillar array possessed sufficient mechanical strength to withstand polymer plasticization without significant deformation. Furthermore, the ultrasonic plasticized melt exhibited distinct flow behavior as the UA time increased. Consequently, the deformation of the nickel micropillar array did not exhibit a linear relationship with increasing UA time, and at a certain UA time value (such as 0.34 s), the micropillars exhibited minimal or even no deformation. By contrast, the silicon template could not withstand polymer plasticization, resulting in increasingly severe damage with increasing UA time. Therefore, when a template can withstand polymer plasticization, its damage intensity does not necessarily increase with UA time and can be mitigated by optimizing UA time settings. However, if the structure lacks sufficient strength, damage severity escalates with longer UA times. In such cases, process parameters should be reduced to minimize or prevent template failure.

### 3.5. Interaction Mechanism of the Melt with the Template

Based on the measured melt pressure and the observed damage to the nickel micropillar arrays and silicon templates, the interaction mechanism between the melt and the template microstructure during ultrasonic plasticization and melt flow was analyzed for UP, UPP, and UPIM. The distinctions among these processes are detailed below.

**Figure 15:**
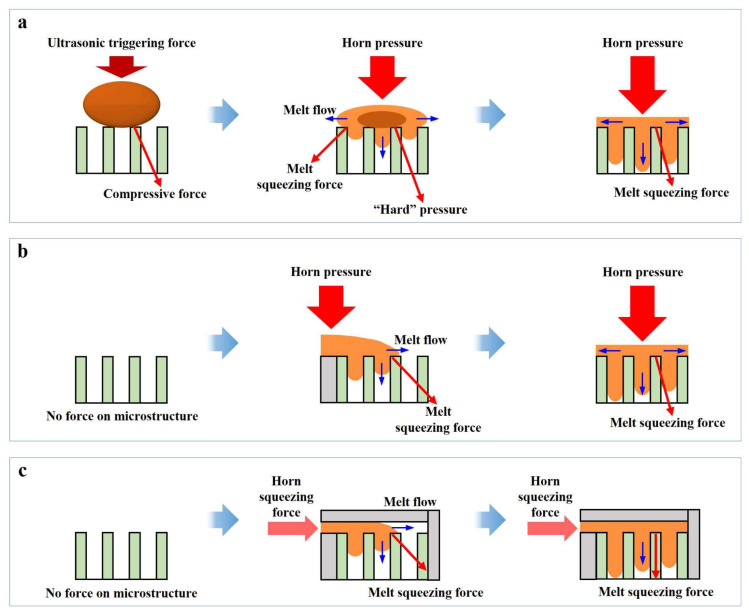
Force mechanisms acting on the template microstructure in UP (**a**), UPP (**b**), and UPIM (**c**).

As illustrated in Figure 15a, the forces acting on the template microstructure in UP can be categorized into three stages. In Stage 1, before the onset of ultrasonic vibration, that is, before the pressure from the ultrasonic sonotrode reaches the triggering threshold, the template microstructure experiences a compressive force from the polymer pellet. This force, induced by the downward pressure of the ultrasonic sonotrode, acts perpendicular to the tangent plane of the pellet at the contact point. Its magnitude depends on the contact conditions between the pellet and the microstructure. In Stage 2, which occurs between the initiation of polymer plasticization and the complete spreading of the melt across the template surface, the microstructure is subjected to two primary forces: a “hard” pressure from the unmelted portion of the pellet and a squeezing force from the flowing melt. As shown in Figure 9a, the “hard” pressure is significantly greater and serves as the dominant factor contributing to deformation or structural damage during this stage. In Stage 3, from the point at which the melt fully covers the upper surface of the template to its solidification, the continued downward motion of the sonotrode induces additional melt flow. During this phase, the melt flows not only downward into the microcavities but also radially outward from the center of the sonotrode. Consequently, the squeezing force acting on the microstructure is deflected from the vertical direction and instead acts along the radial direction of the sonotrode.

To enable a comprehensive comparison of the forces acting on the template microstructures across the three processes, the force evolution in UPP and UPIM was also divided into three stages: (1) before the melt enters the template microstructure, (2) after the melt enters but before it spreads uniformly across the upper surface, and (3) after the melt fully spreads across the upper surface but before solidification. As illustrated in Figure 15b,c, during the first stage, the template microstructures in both UPP and UPIM remain unstressed. In the second stage, they are subjected to a squeezing force generated by the forward flow of the melt. A key distinction is observed at this stage: in UPP, the melt is driven forward by the direct pressure of the ultrasonic sonotrode, whereas in UPIM, melt flow is driven by the squeezing force generated at the sprue by the sonotrode. Consequently, the squeezing force exerted on the template microstructure is greater in UPP than in UPIM, consistent with the results in Figure 10a,b. During the third stage, the force profile in UPP resembles that in UP, but the overall magnitude is lower, as supported by the data in Figure 9 and Figure 10a. By contrast, in UPIM, the melt flows only downward into the microcavities, so the squeezing force on the template microstructure remains aligned with that direction. As shown in Figure 9a and Figure 10a,b, this squeezing force in UPIM is substantially lower than in both UP and UPP during the same stage.

The differences in the melt–template interaction mechanisms among the three processes can be summarized as follows. First, force is applied to the template microstructure earlier in UP than in UPP or UPIM. In UP, stress begins the moment the ultrasonic sonotrode contacts the polymer pellets, whereas in UPP and UPIM, the template is unstressed until the melt enters the microstructure. Second, in UP, plasticization occurs directly above the template, resulting in complex and less controllable force distribution. By contrast, in UPP and UPIM, plasticization takes place away from the template, avoiding direct mechanical or thermal effects on the microstructure during this stage. Third, melt flow in both UP and UPP is driven by direct pressure from the sonotrode, whereas in UPIM, it is driven by a secondary squeezing force from the melt at the sprue. As a result, the force transmitted to the template microstructure is higher in UP and UPP than in UPIM.

### 3.6. Microstructure Performance Analysis and Comparison

The performance of polymer microstructures is critical to their functionality and applicability. To evaluate whether fabrication method influences microstructural performance, the PP rectangular microprisms produced via UP, UPP, and UPIM were assessed with respect to differences in crystalline phase formation, thermal stability (melting point and crystallinity), and mechanical properties (hardness and elastic modulus).

**Figure 16:**
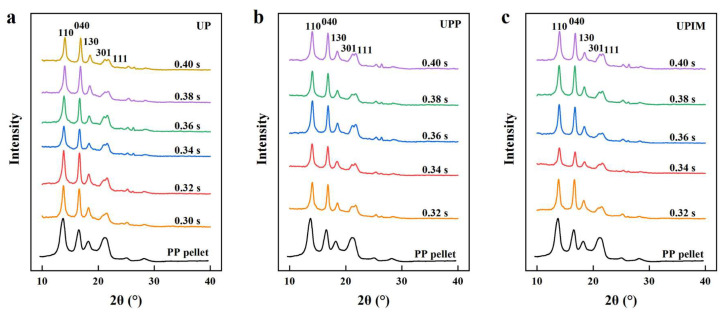
XRD patterns of the PP pellet and the PP rectangular microprisms prepared by UP (**a**), UPP (**b**), and UPIM (**c**), respectively, with different UA times.

As shown in Figure 16a, the PP pellet exhibits four diffraction peaks at 2θ values of 14.1°, 17.0°, 18.52°, and 21.6°, corresponding to the (110), (040), (130), and (111) planes of the α-crystal form, respectively [33,34]. By contrast, PP rectangular microprisms fabricated via UP exhibit an additional diffraction peak at 2θ = 21.3°, attributed to the (301) plane of the β-crystal form [35]. The intensity of this peak increases with longer UA times, indicating that ultrasonic vibration during UP induces a crystal transformation, with the β-phase content increasing as UA time is extended. A similar trend is indicated by Figure 16b,c for samples produced via UPP and UPIM, suggesting that crystal transformation behavior is consistent across all three fabrication methods.

**Figure 17:**
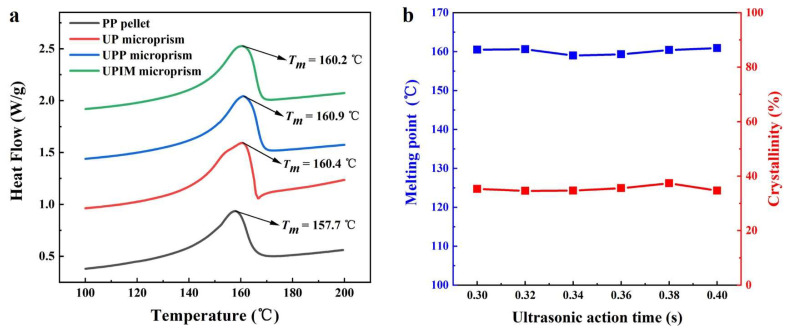
(**a**) DSC curves of the PP pellet and the PP rectangular microprisms prepared by UP, UPP, and UPIM, respectively, at a UA time of 0.38 s. (**b**) Melting point and crystallinity of the PP rectangular microprism prepared by UP versus UA time.

As shown in Figure 17a, the melting points of PP rectangular microprisms fabricated via UP, UPP, and UPIM are approximately 160.4 °C, 160.9 °C, and 160.2 °C, respectively, with corresponding crystallinity values of 37.4%, 37.5%, and 36.0%, calculated using Equation (1). In comparison, the PP pellet exhibits a lower melting point of 157.7 °C and crystallinity of 31.3%. These results demonstrate that ultrasonic vibration plasticization increases both the melting point and crystallinity of the fabricated microstructures relative to the raw material. However, the differences among the three fabrication processes are negligible in terms of thermal stability. As shown in Figure 17b, the melting point varies from 159.0 °C to 160.9 °C, and the crystallinity ranges from 34.6% to 37.4%, neither changing substantially with the UA time. Thus, the UA time had minimal effect on the thermal properties of the PP microstructures fabricated via UP or the applied UA time range was insufficient to induce notable changes.

**Figure 18:**
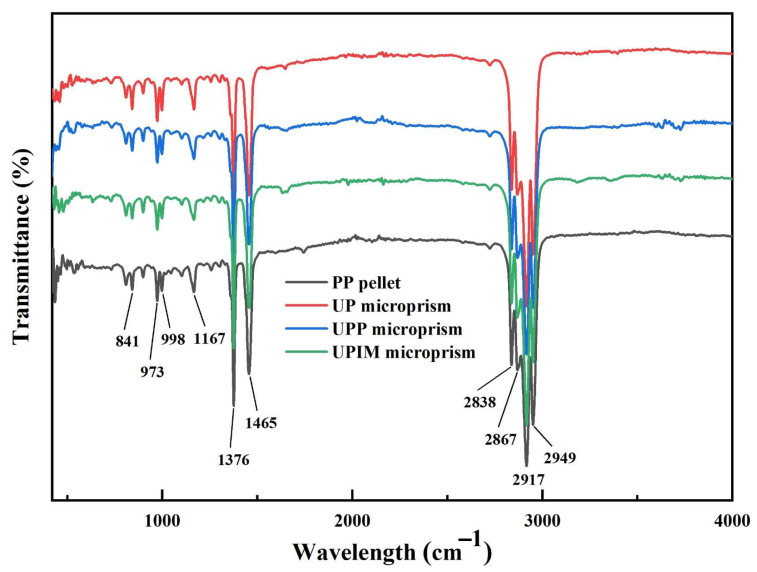
FTIR spectra of the PP pellet and PP rectangular microprisms fabricated via UP, UPP, and UPIM at a UA time of 0.40 s.

As shown in Figure 18, the FTIR spectra of the PP pellet exhibit characteristic peaks at 2949 cm^−1^, 2917 cm^−1^, 2867 cm^−1^, 2838 cm^−1^, 1456 cm^−1^, 1376 cm^−1^, 1167 cm^−1^, 998 cm^−1^, 973 cm^−1^, and 841 cm^−1^ [36]. These same peaks are present in the rectangular microprisms fabricated through UP, UPP, and UPIM, indicating no change in chemical composition or molecular vibrational structure. This result suggests that although the transient temperature of the PP melt during ultrasonic plasticization may exceed the oxidative pyrolysis threshold, the chemical integrity of the PP microprisms remains preserved. During ultrasonic plasticization molding, the melt temperature rapidly increases and decreases, with the melt remaining in a high-temperature state only momentarily. This may explain why oxidative decomposition did not occur even when the melt temperature reached as high as 478 °C.

**Figure 19:**
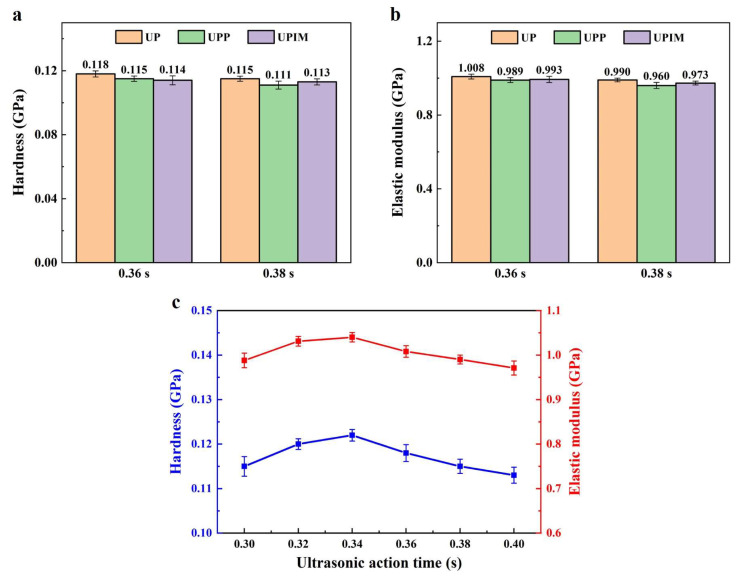
(**a**) Hardness and (**b**) elastic modulus of PP rectangular microprisms fabricated via UP, UPP, and UPIM at UA times of 0.36 s and 0.38 s (**c**) Hardness and elastic modulus of PP rectangular microprisms fabricated via UP versus UA time. Error bars represent the standard deviation of measurements for each group.

As shown in Figure 19a, hardness differences among PP rectangular microprisms fabricated via UP, UPP, and UPIM at identical UA times are minimal. For UA times of 0.36 s and 0.38 s, the difference between maximum and minimum hardness values is only 3.51% and 3.60% of the minimum value, respectively. A similar trend is indicated by Figure 19b for the elastic modulus. These results indicate that under identical processing conditions, hardness and elastic modulus remain largely consistent across all three fabrication methods. As shown in Figure 19c, increasing UA time during UP initially increases both hardness and elastic modulus, followed by a subsequent decline. These two parameters vary synchronously. With increasing UA time, higher melt temperatures improve flowability and microcavity filling, enhancing mechanical performance. However, excessive heating weakens intermolecular interactions at the surface, resulting in reduced hardness and elastic modulus.

Overall, the PP microstructures fabricated through UP, UPP, and UPIM exhibit no significant performance differences within the parameters investigated. Although differences in the location of raw material plasticization and whether the ultrasonic sonotrode action surface encompasses the microcavity account for the differences in melt fluidity and microstructure formation among the three processes, ultimately, PP pellets were plasticized under identical process conditions. The heat generation effect and molecular chain disentanglement and decomposition effect induced by ultrasonic vibration were essentially consistent. This may be the primary reason why no significant differences in thermal stability and mechanical properties were observed in the PP microstructures fabricated through the three processes. Variations in melt flow behavior, depending on whether the template microstructure lies directly beneath the ultrasonic sonotrode, do not lead to notable changes in the thermal stability or mechanical properties of the fabricated structures.

## 4. Conclusions

This study compared three polymer ultrasonic plasticization microstructure molding technologies, namely UP, UPP, and UPIM, through slit flow tests, with UP examined in detail. The key findings are as follows:

(1) Increasing UA time, ultrasonic amplitude, and loading pressure improved microstructure formation and enhanced melt fluidity in all three processes. Melt fluidity was affected by micropore width rather than depth.
(2) Under identical conditions, UP yielded the best microstructure formation and melt fluidity, followed by UPP and UPIM.
(3) Melt flow behavior improved when the microcavity was located within the action area of the ultrasonic sonotrode and directly subjected to axial vibration.
(4) In UP, continuous ultrasonic vibration maintained melt temperature and pressure during micropore filling, thereby sustaining fluidity.
(5) Among the three processes, UPIM caused the least damage to the template microstructures, followed by UPP, while UP resulted in the most severe damage. The template in UP was subjected to most complex and intense forces.
(6) The effect of UA time on template damage in UP depended on template strength; structurally robust templates could be reused by optimizing processing time.
(7) PP microstructures fabricated through UP, UPP, and UPIM exhibited no significant differences in thermal stability or mechanical properties.

## Figures and Tables

**Table 1:** Investigated process parameters and their values.

Group	Raw Material	Ultrasonic Action Time (s)	Ultrasonic Amplitude (μm)	Ultrasonic Loading Pressure (kPa)
Group A	PP	0.30, 0.32, 0.34, 0.36, 0.38, 0.40	60	300
Group B	PP	0.36	40.8, 45.6, 50.4, 55.2, 60	300
Group C	PP	0.36	60	100, 150, 200, 250, 300
Group D	PS	0.28, 0.30, 0.32, 0.34, 0.36, 0.38, 0.40	60	300
Group E	PC	0.42, 0.44, 0.46, 0.48, 0.50, 0.52, 0.54	60	300

**Table 2:** Cross-sectional dimensions of the rectangular microgrooves.

Type	Aspect Ratio	Design Dimension		Actual Dimension	
		Width (µm)	Depth (µm)	Width (µm)	Depth (µm)
Equal-width RMGs (Mold core I)	5:1	200	1000	204.70	1008.44
	10:1	200	2000	200.84	2005.98
	15:1	200	3000	196.98	2992.20
Equal-depth RMGs (Mold core II)	12:1	250	3000	250.93	3002.35
	15:1	200	3000	196.79	2996.32
	20:1	150	3000	146.83	2994.32

**Table 3:** Maximum melt temperatures and temperature differences between the inlet and microgroove interior.

Process	Maximum Melt Temperature at Microgroove Inlet, *T*_max-inlet_ (°C)	Maximum Melt Temperature Inside Microgroove, *T*_max-inside_ (°C)	Difference Between *T*_max-inlet_ and *T*_max-inside_ (°C)
UP	478.8	438.3	40.5
UPP	278.4	254.4	24.0
UPIM	236.9	139.0	97.9

## Data Availability

The original contributions presented in the study are included in the article/Supplementary Material, further inquiries can be directed to the corresponding author.

## Supplementary Materials

The following supporting information can be downloaded at https://www.mdpi.com/article/10.3390/polym17192576/s1, Figure S1: Processing of nickel micropillar arrays by LS-WEDM; Figure S2: Schematic distribution of nanoindentation measurement points; Figure S3: Morphologies of the micropore arrays prepared by UP using the nickel micropillar arrays in Figure 12 as templates; Figure S4: Morphologies of the micropore arrays prepared by UPP and UPIM, respectively, using the nickel micropillar arrays in Figure 13 as templates.
