# Supplementary material for: Characterization and Comparison of Polymer Melt Fluidity Across Three Ultrasonic Plasticization Molding Technologies
Source: Polymers (Basel). 2025 Sep 24;17(19):2576. doi: 10.3390/polym17192576 (PMC12526616; doi:10.3390/polym17192576)
Supplement: Supplementary file 1 [file polymers-17-02576-s001.zip › polymers-3852950-supplementary.pdf]

Supplementary Material

Characterization and comparison of polymer melt fluidity across three ultrasonic plasticization molding technologies

Shiyun Wu[1], Jianjun Du[1], Junfeng Liang[2], Likuan Zhu[2], Jianguo Lei[2,*]

[1]*School of Mechanical Engineering and Automation, Harbin Institute of Technology (Shenzhen), Shenzhen 518055, China*

[2]*College of Mechatronics and Control Engineering, Shenzhen University, Shenzhen 518061, China*

[*]**Corresponding author. E-mail address:** ljg_sc111@163.com.

Supplementary Figure 1

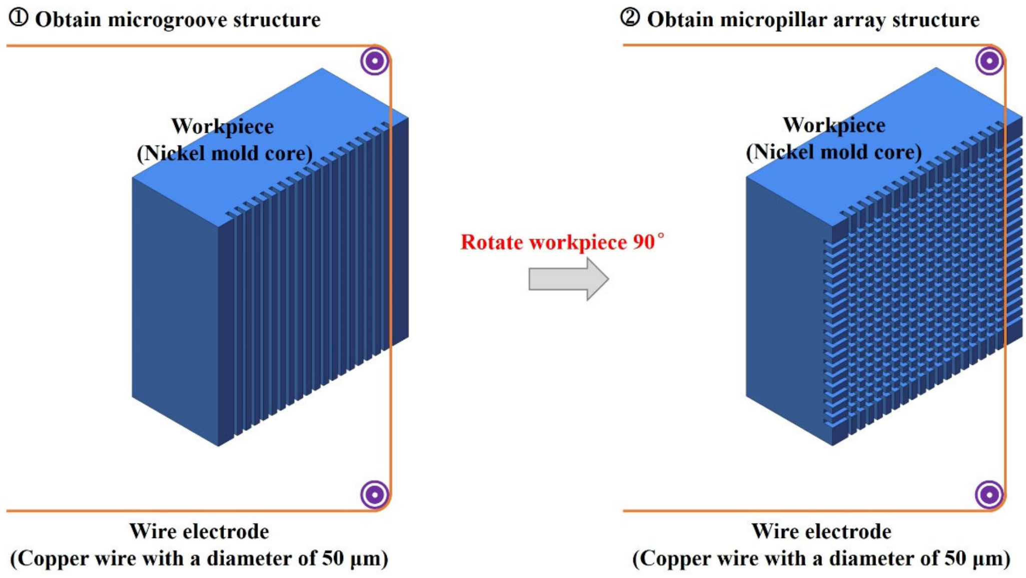

Figure S1. Processing of nickel micropillar arrays by LS-WEDM.

Supplementary Figure 2

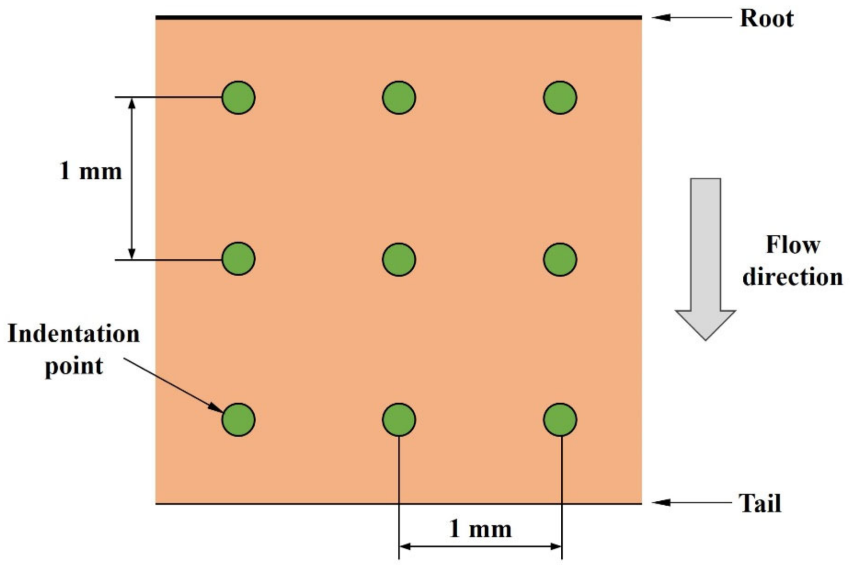

Figure S2. Schematic distribution of nanoindentation measurement points.

Supplementary Figure 3

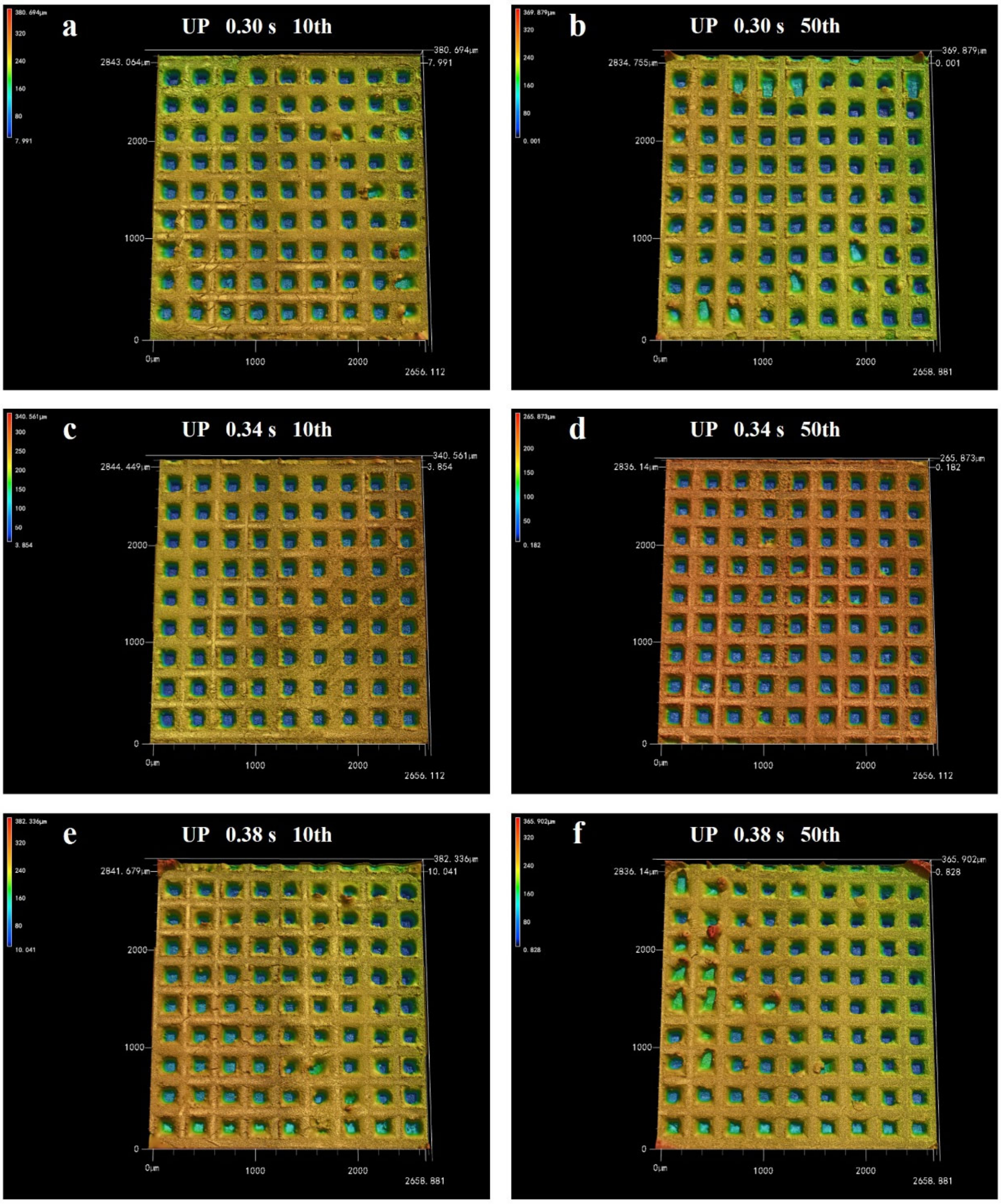

Figure S3. Morphologies of the micropore arrays prepared by UP using the nickel micropillar arrays in Figure 12 as templates: (a) corresponds to Figure 12(b), (b) corresponds to Figure 12(c), (c) corresponds to Figure 12(f), (d) corresponds to Figure 12(g), (e) corresponds to Figure 12(j), and (f) corresponds to Figure 12(k).

Supplementary Figure 4

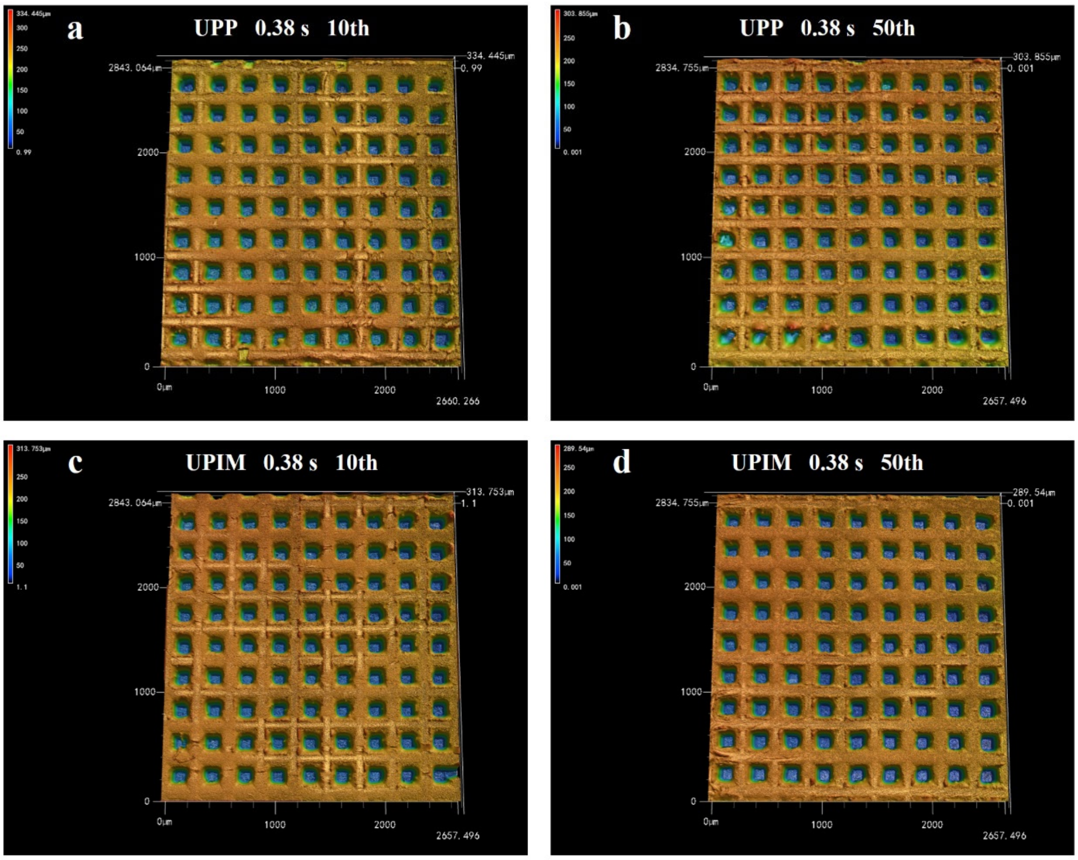

Figure S4. Morphologies of the micropore arrays prepared by UPP and UPIM, respectively, using the nickel micropillar arrays in Figure 13 as templates: (a) corresponds to Figure 13(b), (b) corresponds to Figure 13(c), (c) corresponds to Figure 13(f), and (d) corresponds to Figure 13(g).
